# High-Sensitivity Cuboid Interferometric Fiber-Optic Hydrophone Based on Planar Rectangular Film Sensing

**DOI:** 10.3390/s20226422

**Published:** 2020-11-10

**Authors:** Wenrui Wang, Yeye Pei, Lingyun Ye, Kaichen Song

**Affiliations:** 1 College of Aeronautics and Astronautics, Zhejiang University, No. 38 Zheda Road, Hangzhou 310027, China; wangwenrui@zju.edu.cn (W.W.); kcsong@zju.edu.cn (K.S.); 2 College of Biomedical Engineering and Instrument Science, Zhejiang University, No. 38 Zheda Road, Hangzhou 310027, China; peiyeye@zju.edu.cn

**Keywords:** fiber-optic hydrophone, phase sensitivity, underwater acoustic detection

## Abstract

Interferometric fiber-optic hydrophones are an important means in the field of underwater acoustic detection. The design of the hydrophone sensor head is the key technology related to its detection sensitivity. In this paper, a high-sensitivity cuboid interferometric fiber-optic hydrophone based on planar rectangular film sensing is proposed, and the sensitivity of the sensor is compared with that of the widely used air-backed mandrel hydrophone under the same conditions. The acoustic characteristic models of the two types of sensors were established by theoretical calculation and simulation analysis to obtain the theoretical pressure sensitivity. Some experiments were performed to examine the theory and design. According to the experiment results, the mean phase sensitivity of the mandrel type was −112.85 dB re 1 rad/μPa in the operating frequency range of 10–300 Hz, and that of the cuboid type was −84.50 dB re 1 rad/μPa. The latter was 28.35 dB higher than the former was. These results are useful for improving hydrophone sensitivity.

## 1. Introduction

Underwater acoustic-sensing technology is the main means of ocean target detection and underwater acoustic communication. In an underwater acoustic-sensing system, a hydrophone is the basic and most important device to detect underwater acoustic signals. Since its appearance in the 1970s, the fiber-optic hydrophone gradually replaced the traditional piezoelectric hydrophone as a popular research topic in the field of underwater acoustic sensing due to advantages such as high sensitivity, large dynamic range, multiplexing capability, and immunity to electromagnetic interference [1,2,3,4]. Most fiber-optic hydrophones operate in the range of tens to thousands of Hertz [5]. However, there has been an increasing number of reports on low-frequency hydrophones in recent years, such as multigas detection [6], seismic exploration [7,8], and oi-pipeline protection [9]. The benefits of low-frequency acoustic detection are increasingly recognized: lower distance-related attenuation and longer propagation distance [10]. The downside is the higher noise level existing in a low-frequency range compared with that in a high-frequency range. Acoustic sensitivity and system noise floor are key to hydrophone research since they determine whether the system can detect effective hydrophone signals in complex underwater environments [11].

The interferometric fiber-optic hydrophone is most widely used [12]. According to different application scenarios, various sensitivity-enhancement techniques have been proposed to improve the acoustic-pressure sensitivity of the hydrophone sensor head. In other words, to increase the phase difference between the two arms of the interferometer caused by the sensing fiber under the same level of sound pressure. Methods can be divided into the three following types: (1) Technology in terms of materials [13,14]. The sensing fiber and elastic material are fixed together in different ways, such as coating, and adhesive and sealing. Material characteristics are optimized to improve the elastic strain according to Hooke’s law. (2) Technology in terms of shape. In addition to the optimization of material properties, a change in elastomer geometry can also improve elastic deformation. According to the different shapes of the elastomer, it can be further divided into mandrel [15,16], planar, and cavity [17] shapes. (3) Technology in terms of structure, including the push–pull [18], air-backed [19], and multilayer-superposition [20] types. The air-backed mandrel type was identified as one of the most useful and widely used hydrophones [21]. From a geometrical point of view, a planar rectangular film is easier to deform than an axisymmetric cylinder is under the same amount of pressure. Therefore, the air-backed planar-type hydrophone with a fiber wound may provide higher sensitivity compared with that of the mandrel type, but relevant research has rarely been reported. In this paper, we propose a high-sensitivity and low-frequency cuboid-type interferometric fiber-optic hydrophone based on air-backed planar rectangular film sensing. Four planar rectangular films and four quarter cylinders form a rounded cuboid structure with sensing fibers wound around the sides of the structure. Sensitivity performance is compared with that of the common mandrel type under the same sensing conditions. Acoustic characteristics of the two types mentioned above were modeled, theoretically analyzed, and experimentally verified. The results of the experiment showed that the mandrel type had an average phase sensitivity of −112.85 dB re rad/μPa in the operating frequency range 10 to 300 Hz, while the mean value of the proposed cuboid type was −84.50 dB re rad/μPa, which was about 28.35 dB higher than the former.

## 2. Theory

### 2.1. General Principle

**Figure 1:**
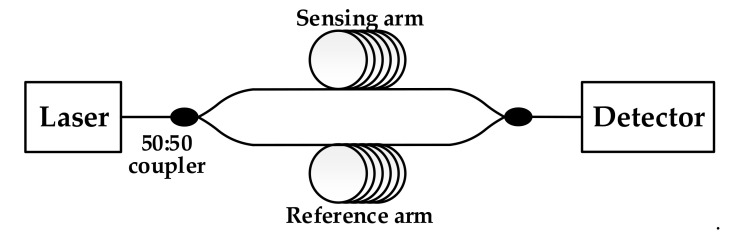
Configuration of fiber-optic Mach–Zehnder interferometer hydrophone.

The interferometric fiber-optic hydrophone is based on optical interferometers, such as Mach–Zehnder, Michelson, and Sagnac interferometers. The schematic diagram configuration of a fiber-optic Mach–Zehnder interferometer hydrophone is illustrated in Figure 1. The effect of sound pressure on the sensitive surface of the sensing arm generates the change of phase difference between the two arms, and light intensity changes that it causes can be detected by the photoelectric detector [22]. After data processing, the original acoustic signal can be obtained.

The conversion of the change of sound pressure to phase difference is characterized by the phase-sensitivity index, which reflects the sensing capability of the hydrophone sensor head. It is defined as the ratio of phase difference between the two arms of the interferometer and the actual sound pressure acting on the sensing surface. Sensitivity index $M_{\phi }$ can be expressed as

(1) $$M_{\phi }=\frac{\varphi_{M}}{P}\;\mathrm{rad}/\mu \mathrm{Pa},$$

where P is the sound pressure, $\varphi_{M}$ is the phase difference, $\varphi_{M}=\frac{2\pi nl\upsilon }{c}$, c is the speed of light in a vacuum, n denotes the refractive index, l is the length of the sensing fiber, and υ is the frequency of light. Hence, phase difference changes can be given by

(2) $$\Delta \varphi =\;\frac{2\pi nl\upsilon }{c}\left(\frac{\Delta n}{n}+\frac{\Delta l}{l}+\frac{\Delta \upsilon }{\upsilon }\right).$$

These three terms, respectively, represent changes caused by the effective refractive index, the physical dimension of the fibers, and the optical frequency jitter. Phase change in interferometric hydrophones is dominantly due to the first two items [23].

### 2.2. Theory Model

#### 2.2.1. Mandrel Type

**Figure 2:**
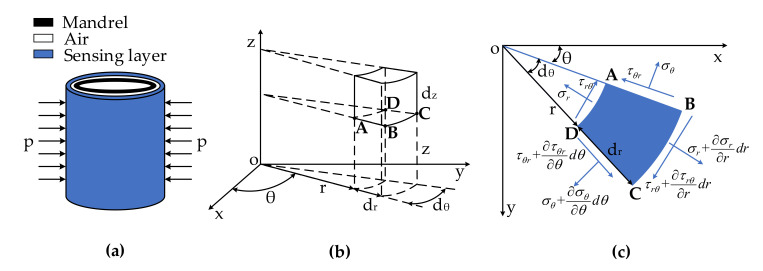
Theoretical modeling of mandrel hydrophone sensor head. (**a**) Pressure model; (**b**) hexahedral element in cylindrical coordinates; (**c**) stress distribution on four sides of hexahedral element in polar coordinates.

As shown in Figure 2, a theoretical sound pressure model of the mandrel type was established in cylindric coordinates. The sensor-head cylinder was divided into three layers. Layer1 is the sensing layer, including the sensing fiber and elastomer; Layer3 is the mandrel structure, as shown in Figure 2a. The sensing layer was subjected to sound pressure p. In the low-frequency sound field (10–300 Hz), the sound wave was much longer than the sensor-head size was, so sound pressure p could be regarded as the radial uniform pressure acting on the cylinder surface.

A hexahedral element is taken from the sensing layer. On the basis of the stress condition in Figure 2b,c, the equilibrium differential equation, geometric equation, and material constitutive model of the microelement could be obtained. By solving simultaneous equations, radial displacement $u_{r}$ can be expressed as [24,25,26]:

(3) $$\frac{\partial u_{r}^{2}}{\partial r^{2}}+\frac{1}{r}\frac{\partial u_{r}}{\partial r}-\frac{u_{r}}{r^{2}}=0.$$

The general solution is $u_{r}=Ar+B\frac{1}{r}$, where A and B are constants.

Suppose that the inner and outer radii of Layer1 are $R_{1}$ and $R_{2}$, respectively, and the inner surface of layer1 is not applied to pressure because of air-filled Layer2. Thus, the boundary condition is

(4) $$\left\{\begin{array}{l} \sigma_{r\left|r=R_{1}\right.}=0\\ \sigma_{r\left|r=R_{2}\right.}=-p \end{array}\right..$$

On the basis of $u_{r}=Ar+B\frac{1}{r}$, A and B can be determined by (3) and (4).

(5) $$\left\{\begin{array}{c} A=\frac{\mu pR_{2}^{2}}{\lambda \left(R_{1}^{2}-R_{2}^{2}\right)}\\ B=\frac{pR_{1}^{2}R_{2}^{2}}{2G\left(R_{1}^{2}-R_{2}^{2}\right)} \end{array}\right.,$$

where λ and G are the Lamé constants, $\lambda =\frac{\mu E}{\left(1+\mu \right)\left(1-2\mu \right)}$, and $G=\frac{E}{2\left(1+\mu \right)}$ [25].

According to Equation (2), the phase difference change between the two arms of the mandrel-type hydrophone sensor head under sound pressure p is [10,24]:

(6) $$\begin{array}{l} \Delta \varphi_{\mathrm{Mandrel}}=\frac{2\pi n}{\lambda_{\nu }}\Delta l\left(1-P_{c}\right)=\frac{2\pi nN}{\lambda_{\nu }}\cdot 2\pi u_{r\left|r=R\right.}\left(1-P_{c}\right)\\ =\frac{4\pi^{2}nN}{\lambda_{\nu }}\left(1-P_{c}\right)\left(\frac{\mu pR_{2}^{2}R}{\lambda \left(R_{1}^{2}-R_{2}^{2}\right)}+\frac{pR_{1}^{2}R_{2}^{2}}{2RG\left(R_{1}^{2}-R_{2}^{2}\right)}\right) \end{array}$$

where $\lambda_{\nu }$ is the optical light wavelength, N is the optical-fiber number of turns, and $P_{c}$ is the photoelastic coefficient [27]:

(7) $$P_{c}=\frac{1}{2}n^{2}\left((1-\mu_{f})P_{12}-\mu_{f}P_{11}\right),$$

where $\mu_{f}$ is Poisson’s ratio of the fiber, $P_{11}$ and $P_{12}$ are Pockels’ coefficients.

#### 2.2.2. Cuboid Type

**Figure 3:**
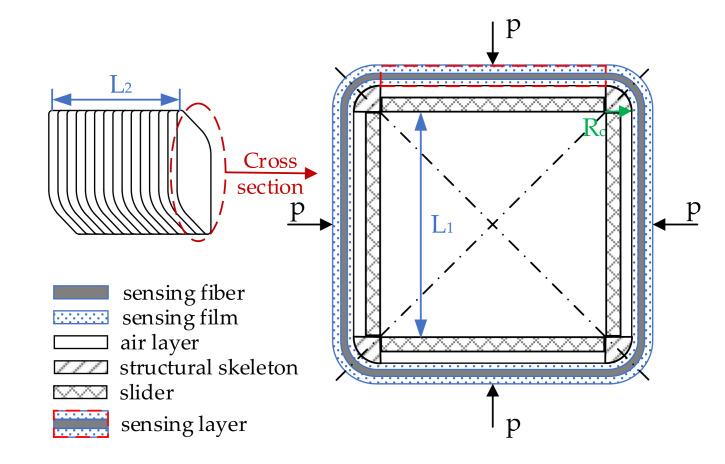
Structure of proposed cuboid-type hydrophone.

Figure 3 is the structure diagram of the cuboid-type sensor head. As shown in the picture, four stainless-steel quarter cylinders make up the structural skeleton, and four stainless-steel sliders are embedded into the sides of the skeleton. The relative position of the slides can be adjusted to leave a gap of 1 mm and form four cuboid layers. The sensing fiber and films are wrapped around the outer side of the skeleton in the order of “film–fiber–film”. After some inflation and seal measures, the four cuboid layers are filled with air. The air layers are connected with each other by some inner holes, and can be inflated and deflated according to the external pressure. Therefore, four air-backed rectangular sensing layers are formed.

**Figure 4:**
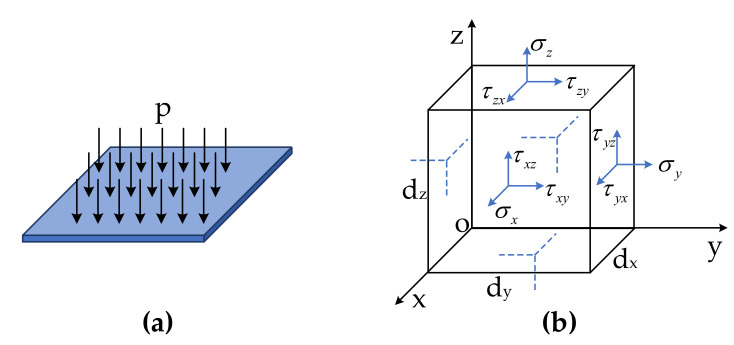
Theoretical modeling of cuboid hydrophone sensor head. (**a**) Pressure model; (**b**) stress distribution of hexahedral element in Cartesian coordinates.

As shown in Figure 4, the theoretical sound pressure model of the cuboid-type was is established in Cartesian coordinates. A hexahedral element was taken from one side of the sensing layer, and the stress condition is shown in Figure 4b. Similarly, sound pressure p can be considered as a uniform load applied on the rectangular surface. 

Suppose that the X-axis length of the sensing surface is L_1,_ and Y-axis length is L_2_; the boundary condition is

(8) $$\left\{\begin{array}{c} {\left(w\right)}_{x=0}=0,{\left(\frac{\partial^{2}w}{\partial x^{2}}\right)}_{x=0}=0\\ {\left(w\right)}_{x=L_{1}}=0,{\left(\frac{\partial^{2}w}{\partial x^{2}}\right)}_{x=L_{1}}=0 \end{array}\right..$$

Displacement w does not have relationship with z, and it can be represented as $w\left(x,y\right)$. The fourth-order differential equation of displacement function $w\left(x,y\right)$ can be obtained as follows [28]:

(9) $$\frac{Ed^{3}}{12(1-\mu^{2})}\left(\frac{\partial^{4}w\left(x,y\right)}{\partial x^{4}}+2\frac{\partial^{4}w\left(x,y\right)}{\partial x^{2}\partial y^{2}}+\frac{\partial^{4}w\left(x,y\right)}{\partial y^{4}}\right)=p.$$

The solution can be expressed in trigonometric series form:

(10) $$w\left(x,y\right)=\frac{48pL_{1}{}^{4}(1-\mu^{2})}{\pi^{5}Ed^{3}}\sum_{m=1,3,5,\cdots }^{\infty }\left(\frac{1}{m^{5}}\right)\left(1-\frac{2+\alpha_{m}\tanh\alpha_{m}}{2\cosh\alpha_{m}}\cosh\frac{2\alpha_{m}y}{L_{2}}+\frac{\alpha_{m}}{2\cosh\alpha_{m}}\frac{2y}{L_{2}}\sinh\frac{2\alpha_{m}y}{L_{2}}\right)\sin\frac{m\pi x}{L_{1}},$$

where $\alpha_{m}=\frac{m\pi L_{2}}{2L_{1}}$.

The maximal value is at the center of the rectangular sensing surface [28]:

(11) $$w{\left(x,y\right)}_{\left|x=\frac{L_{1}}{2},y=0\right.}=\frac{5pL_{1}^{4}(1-\mu^{2})}{32Ed^{3}}.$$

It is very complicated to calculate the total deformation value of the sensing fiber by analytical solution. The phase sensitivity of the cuboid-type hydrophone is further evaluated by numerical simulation.

The analysis model of the cuboid-type hydrophone sensor head was established in COMSOL Multiphysics software using an Acoustic-Solid Interaction Multiphysics coupling. This coupling involved two physics interfaces, the Pressure Acoustics and Solid Mechanics modules. The sensor head was immersed in a 1 m diameter circular water domain with an incident plane wave applied on the domain boundary from the surroundings. The inside and outside surfaces of the sensing layer between acoustic domain and solid were automatically established via the Acoustic–Structure Boundary coupling feature. This condition ensured continuity in both pressure and acceleration on the boundary.

The mesh of the model was generated by the physics-controlled sequence mode, and the average value of the element qualities was 0.7605. The maximal and minimal mesh size of the model were 67 and 0.3 mm, respectively, resolving the acoustic wavelength in the model well.

**Figure 5:**
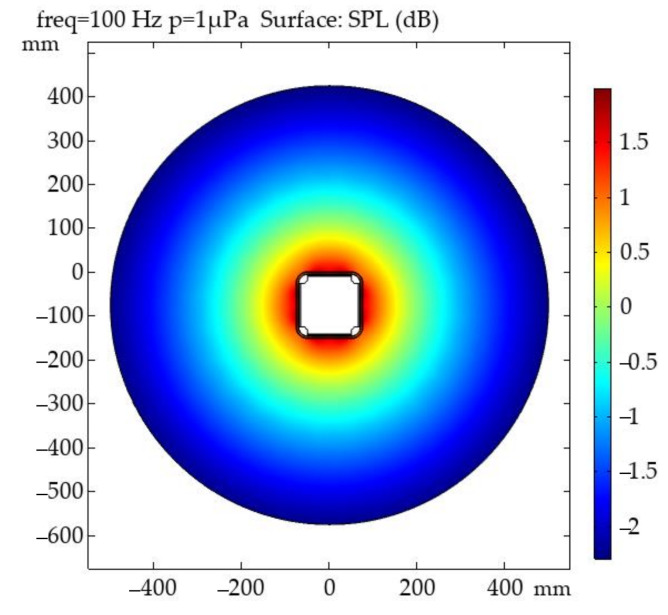
Sound pressure level (dB re 1 μPa) in model’s acoustic domain.

Figure 5 illustrates the geometry of the 2D model, and the structure size of the sensor head was the same as that shown in Figure 3. Sound pressure level (SPL) in the acoustic domain (both water and air domain) is also displayed in Figure 5.

**Figure 6:**
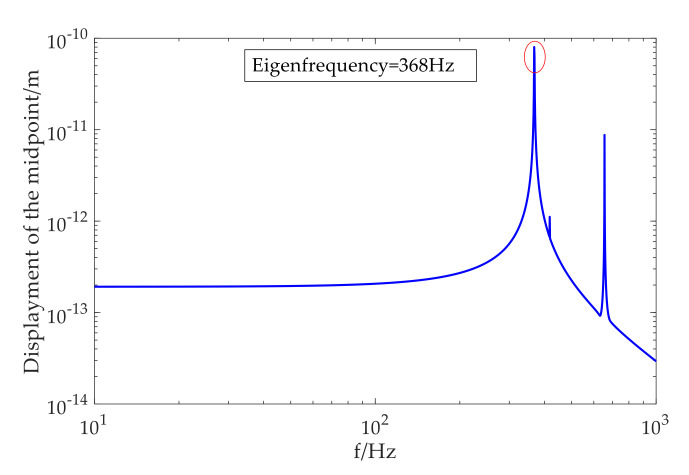
Simulation frequency response of cuboid-type hydrophone in range 10–1000 Hz.

The model was solved using a frequency-domain sweep. The frequency sweep resulted in a frequency response where the displacement was evaluated at the midpoint of the sensing layer plane, as shown in Figure 6. The first vibration mode in the frequency sweep appeared at 368 Hz, and this eigenfrequency was the result of structure–design optimization to match the detection frequency range 10–300 Hz. The cuboid-type hydrophone was predicted to have a flat response for this designed frequency band.

**Figure 7:**
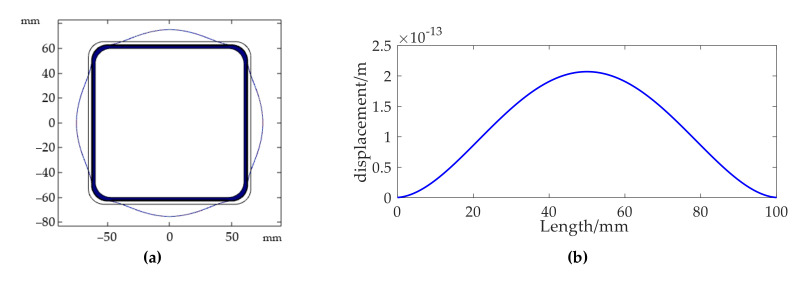
(**a**) Deformation displayed by zoomed-in displacement; (**b**) detailed displacement curve.

Displacement along the normal direction of the sensing surface at 100 Hz is illustrated in Figure 7. The maximal ordinate value of the curve shown in Figure 7b was approximately value $w_{\max}$ calculated in Equation (11). According to the formula, maximal displacement was calculated as 1.994 × 10^−13^ m, and the simulation value was 2.067 × 10^−13^ m. The simulated and theoretical agreed well, and the accuracy of the finite-element solution could thus be guaranteed. 

The fiber-length change due to sound pressure could be regarded as the length difference before and after fiber deformation. In other words, the total variation of fiber length could be estimated by

(12) $$\Delta l=4N(L_{d}-L_{1}),$$

where $L_{d}$ is curve length in Figure 7b.

The phase difference of the cuboid-type was

(13) $$\varphi_{Cuboid}=\frac{2\pi n}{\lambda_{\nu }}\Delta l\left(1-P_{c}\right).$$

### 2.3. Theoretical-Calculation Result

The design parameters of the two developed hydrophones are shown in Table 1. On the basis of the parameters and Equations (6) and (7), the theoretical phase sensitivity of the mandrel-type hydrophone $M_{\varphi \_Mandrel}$ could be determined as

(14) $$M_{\varphi \_Mandrel}=20\mathrm{lg}\left(\frac{\frac{\varphi_{Mandrel}}{p}}{M_{r}}\right)=-108.03\;\mathrm{dB}\;\mathrm{re}\;1\mathrm{rad}/\mu \mathrm{Pa}.$$

On the basis of the simulation results in Figure 6 and Equations (12) and (13), the average theoretical phase sensitivity of cuboid-type $M_{\varphi \_\mathrm{Cuboid}}$ could be obtained as

(15) $$M_{\varphi \_\mathrm{Cuboid}}=20\mathrm{lg}\left(\frac{\frac{\varphi_{Cuboid}}{p}}{M_{r}}\right)=-81.47\;\mathrm{dB}\;\mathrm{re}\;1\mathrm{rad}/\mu \mathrm{Pa}.$$

The above calculation results show that the theoretical phase sensitivity of the cuboid type was 26.56 dB higher than that of the mandrel type under the same material parameters and sensitive area.

## 3. Experiments

### 3.1. Experiment Methods

**Figure 8:**
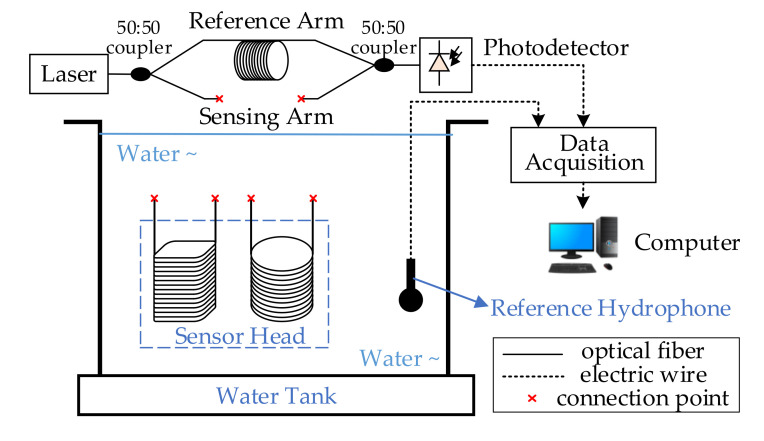
Measurement setup.

The configuration of the phase-sensitivity measurement scheme shown in Figure 8 was implemented to testify the theory and design. The two developed hydrophone sensor heads and a reference piezoelectric hydrophone SA3016-07-02 (sensitivity −168dB re 1V/μPa, made by Hangzhou Applied Acoustics Research Institute, Hangzhou, China) were placed on the same plane in the water tank. The water tank was used to provide a low-frequency (10–300 Hz) test environment. 

When an acoustic signal was generated in the water tank, sound pressure was measured by the piezoelectric hydrophone. The horizontal distance between the piezoelectric-hydrophone probe and the two developed hydrophone sensor heads was much smaller than the acoustic wavelength was, so that the detected sound pressure by the piezoelectric hydrophone could be regarded as the local pressure applied on the sensing surface of the sensor heads.

The proposed hydrophone system was based on the Mach–Zehnder interferometer (MZI), which consisted of a DFB laser (sc-Lightsource Ltd, Mianyang, China, 1550 nm, 10mW), photodetector (Beijing Lightsensing Technologies Ltd, Beijing, China, LSIPD LD-50, responsivity 0.85 mA/mW), reference arm, sensing arm, and two 50:50 couplers, as shown in Figure 8. All used optical devices and fiber were polarization-maintaining. The two developed sensor heads could, respectively, connect with the sensing arm of the interferometer. Therefore, the sensitivity test was performed under the same experiment conditions.

The laser was divided into two beams by the 50:50 coupler, passing through the sensing arm and the reference arm of the MZI, respectively. Interference occurred when the beams were combined by the other coupler. When the developed sensor heads were exposed to sound pressure, the length of the wrapped fiber changed due to the deformation of the sensing layer. Combined with the optical-fiber photoelastic effect, the phase of transmitting light in the sensing arm accordingly changed, leading to fluctuation of relative interference intensity. Signals were collected by data-acquisition card PXIe-1078 (National Instruments, Austin, TX, USA) after photoelectric conversion by the photoelectric detector. Collected data were sent to the computer for demodulation processing; thus, phase difference information was obtained.

On the basis of the detected sound pressure level and phase difference, acoustic sensitivity could be calculated according to Equation (1). 

### 3.2. Experiment Results

**Figure 9:**
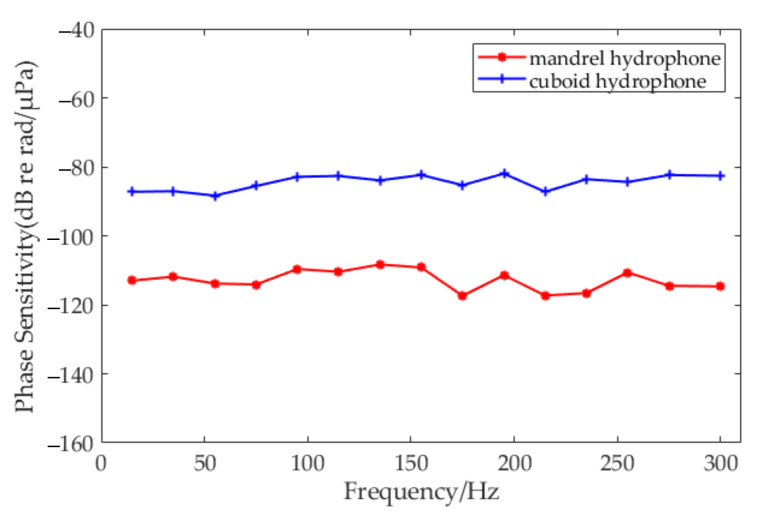
Frequency response of two developed hydrophones in 10–300 Hz range.

As shown in Figure 9, experiment results indicated that the average phase sensitivity of the mandrel-type sensor head was −112.85dB re 1 rad/μPa in the operating frequency range from 10 to 300 Hz, which differed from the theoretical value by 4.82 dB. The average phase sensitivity of the proposed cuboid-type was −84.50 dB re 1 rad/μPa, which was 3.03 dB different from the theoretical value. Compared with the former, improvement was about 28.35 dB.

**Figure 10:**
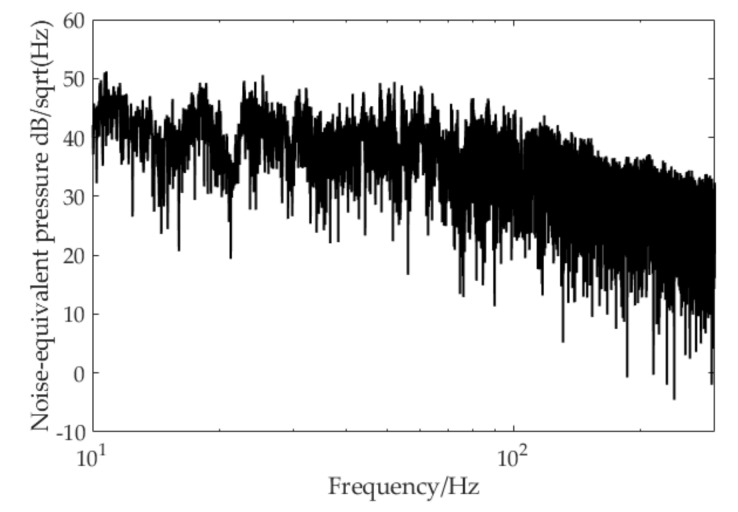
Noise-equivalent pressure level of cuboid-type hydrophone.

The noise-equivalent pressure was evaluated in the experiment without an acoustic signal input, and results from 10 to 300 Hz are illustrated in Figure 10. It can also be considered as the minimal detectable sound pressure level or the resolution of the proposed hydrophone system. This index was calculated by system phase noise floor and phase sensitivity via Equation (15). As shown in the figure, the minimal detectable sound pressure level achieved by the hydrophone system was about 35 to 50 dB in the designed detection band, which was lower than the DSS0 level is [19].

## 4. Conclusions

In summary, we presented a high-sensitivity and low-frequency (10–300Hz) cuboid-type interferometric fiber-optic hydrophone based on air-backed planar rectangular film sensing. Phase sensitivity was both theoretically and experimentally evaluated, and it showed good correlation according to the results. The mean value of its experimental phase sensitivity was about –84.50 dB re 1 rad/1μPa, representing an improvement in sensitivity by 28.35 dB compared to the common mandrel-type hydrophone. The proposed cuboid hydrophone provides a useful means for improving the sensitivity performance of interferometric fiber-optic hydrophones.

## Figures and Tables

**Table 1:** Parameters used in theoretical phase-sensitivity analysis.

Property.	Value
Refractive index of fiber	n = 1.45
Number of fiber turns	N = 400
Pockels’ coefficients of fiber	$P_{11}$ = 0.116, $P_{12}$ = 0.255
Poisson’s ratio of fiber	$\mu_{f}$ = 0.17
Wavelength	$\lambda_{\nu }$ = 1550 nm
Sound pressure level	0 dB re 1V/μPa
Acoustic signal frequency range	10–300 Hz
Young’s modulus of equivalent sensing layer	E = 4GPa
Poisson’s ratio of equivalent sensing layer	μ = 0.45
Thickness of equivalent sensing layer	d = 2.5 mm
Inner radius of mandrel type	$R_{1}$ = 63.5 mm
Outer radius of mandrel type	$R_{2}$ = 66 mm
Deformation radius of mandrel type	R = 64.5 mm
Width of cuboid type sensing layer	$L_{1}$ = 100 mm
Length of cuboid type sensing layer	$L_{2}$ = 140 mm
Cylinder skeleton radius of cuboid type	$R_{c}$ = 20 mm
